# Impact of environmental hygiene interventions on healthcare-associated infections and patient colonization: a systematic review

**DOI:** 10.1186/s13756-022-01075-1

**Published:** 2022-02-19

**Authors:** Alexandra Peters, Marie N. Schmid, Pierre Parneix, Dan Lebowitz, Marlieke de Kraker, Julien Sauser, Walter Zingg, Didier Pittet

**Affiliations:** 1 Infection Control Programme and WHO Collaborating Center on Patient Safety, University of Geneva Hospitals and Faculty of Medicine, 4 Rue Gabrielle-Perret-Gentil, 1211 Geneva 14, Switzerland; 2 University of Geneva, Geneva, Switzerland; 3 Nouvelle Aquitaine Healthcare-Associated Infection Control Centre, Bordeaux University Hospital, Bordeaux, France; 4 Department of Infectious Diseases and Hospital Epidemiology, University Hospital of Zurich, Zurich, Switzerland

**Keywords:** Cleaning, Disinfection, Infection prevention, Healthcare-associated infection, Healthcare environmental hygiene, Infection control, Environmental services, Intervention

## Abstract

**Background:**

Healthcare-associated infections (HAI) are one of the gravest threats to patient safety worldwide. The importance of the hospital environment has recently been revalued in infection prevention and control. Though the literature is evolving rapidly, many institutions still do not consider healthcare environmental hygiene (HEH) very important for patient safety. The evidence for interventions in the healthcare environment on patient colonization and HAI with multidrug-resistant microorganisms (MDROs) or other epidemiologically relevant pathogens was reviewed.

**Methods:**

We performed a systematic review according to the PRISMA guidelines using the PubMed and Web of Science databases. All original studies were eligible if published before December 31, 2019, and if the effect of an HEH intervention on HAI or patient colonization was measured. Studies were not eligible if they were conducted in vitro, did not include patient colonization or HAI as an outcome, were bundled with hand hygiene interventions, included a complete structural rebuild of the healthcare facility or were implemented during an outbreak. The primary outcome was the comparison of the intervention on patient colonization or HAI compared to baseline or control. Interventions were categorized by mechanical, chemical, human factors, or bundles. Study quality was assessed using a specifically-designed tool that considered study design, sample size, control, confounders, and issues with reporting. The effect of HEH interventions on environmental bioburden was studied as a secondary outcome.

**Findings:**

After deduplication, 952 records were scrutinized, of which 44 were included for full text assessment. A total of 26 articles were included in the review and analyzed. Most studies demonstrated a reduction of patient colonization or HAI, and all that analyzed bioburden demonstrated a reduction following the HEH intervention. Studies tested mechanical interventions (n = 8), chemical interventions (n = 7), human factors interventions (n = 3), and bundled interventions (n = 8). The majority of studies (21/26, 81%) analyzed either *S. aureus*, *C. difficile*, and/or vancomycin-resistant enterococci. Most studies (23/26, 88%) reported a decrease of MDRO-colonization or HAI for at least one of the tested organisms, while 58% reported a significant decrease of MDRO-colonization or HAI for all tested microorganisms. Forty-two percent were of good quality according to the scoring system. The majority (21/26, 81%) of study interventions were recommended for application by the authors. Studies were often not powered adequately to measure statistically significant reductions.

**Interpretation:**

Improving HEH helps keep patients safe. Most studies demonstrated that interventions in the hospital environment were related with lower HAI and/or patient colonization. Most of the studies were not of high quality; additional adequately-powered, high-quality studies are needed.

*Systematic registration number*: CRD42020204909

**Supplementary Information:**

The online version contains supplementary material available at 10.1186/s13756-022-01075-1.

## Background

Clean healthcare facilities look appealing, offer a sense of security and increase patient satisfaction [1]. Although visually clean facilities have become the standard of healthcare settings in high-income countries, cleanliness not only plays a role in quality of care, but in its safety. The microbiological aspect of cleanliness, healthcare environmental hygiene (HEH), has remained a neglected field, with little investment beyond what is considered the norm. Few high-quality studies link interventions in HEH to a reduction in either patient colonization with epidemiologically relevant pathogens or healthcare-associated infections (HAI). Though there are many reasons for this, one is the lack of literature critically evaluating the role of HEH in patient safety.

HAI are acquired during hospital stay [2] and cause more deaths worldwide than malaria, tuberculosis, and AIDS combined, and the burden of the six main types of HAI is higher than the total burden of the 32 major communicable diseases [3, 4]. These infections also increase morbidity, prolong hospital stay, and are a major financial burden to healthcare systems [5, 6]. The total annual global cost for five of the most common types of HAI is estimated at $8.3–$11.5 billion [7]. Despite their ubiquity, still much is unknown about how to prevent HAI, and no single hospital or healthcare facility in the world can claim to be unaffected.

While HAIs are usually the result of an infection with the patient’s own flora, this flora can change due to colonization with hospital pathogens through HCWs’ hands or from the hospital environment. Definitively knowing whether an HAI came from the patient’s environment or from another source is difficult. Though it is known that some bacteria are more often transmitted through the patient environment than others, it is comparatively rare that extensive investigations are performed at the time of diagnosis. Usually such investigations are reserved for unusual infections or outbreak situations, in hospitals with sufficient resources to undertake them.

Over the past 25 years, best practice interventions such as hand hygiene in patient care have reduced the number of HAIs [8, 9]. Poor hand hygiene has been recognized as being one of the main drivers of HAIs among patients [9]. Even if such practices can reduce HAIs by up to 50%, there is still a remaining proportion that needs to be addressed and where HEH may play a role [10]. A prerequisite for addressing some of these challenges is to review the literature to evaluate whether HEH interventions have a direct effect on HAI and thus, on patient safety.

HEH is essential for all types of healthcare facilities, from hospitals and long-term care facilities to home care environments. Environmental hygiene builds on both technical and human components, and it includes all aspects of the healthcare environment that are not the patient or the HCWs themselves. The technical component includes cleaning and disinfection of surfaces, water management, air control, waste management, laundry, and sterilization and device reprocessing. The human component includes best practice implementation, staff management, and environmental services departments’ structural organization [11]. This component includes the evaluation of the cost and value of HEH interventions and programs, the training and monitoring of staff, their career development and workflow organization. Both of these components carry major implications for the well-being of patients, HCWs, the community and the larger natural environment.

Beyond the biological plausibility that the healthcare environment has a direct effect on patient safety, a number of reports over the last decades increasingly highlighted the potential impact of environmental hygiene on health [12, 13]. Most common healthcare-associated pathogens are known to survive on surfaces for hours or days, some for weeks and a few for over a year [14, 15]. It has been shown that hygiene failures correlate strongly with HAI in an ICU setting [16]. There is an increase of 150–500% in the chance of acquiring a pathogen if the prior room occupant was colonized with it [17].

This paper reviews the evidence-base for the ability of interventions in the hospital environment to reduce patient colonization with multidrug-resistant microorganisms (MDROs) and other epidemiologically relevant pathogens, and to prevent HAI. This exercise is difficult for a number of reasons. First, high-quality randomized controlled trials in HEH are sparse. Secondly, the bulk of studies are retrospective or prospective before-and-after studies with limited methodological quality. Third, there is heterogeneity of the field about “clean environment” and how environmental hygiene is defined. Finally, HEH interventions are often combined with other infection prevention and control (IPC) interventions such as hand hygiene or a reorganization of patient care. These confounding factors can cause difficulty when determining whether outcomes are a direct effect of an HEH intervention.

## Methods

We performed the systematic review protocol according to the PRISMA checklist [18], in both the PubMed and Web of Science databases. The full search strategies are available in the Additional file 1. The primary outcome is a comparison of the measure of patient colonization or HAI compared to baseline/control. HAI was defined according to the WHO definition [2].

The secondary outcome was environmental bioburden as defined as either cultured environmental samples or adenosine tri-phosphate (ATP) sampling. Although ATP sampling is technically a proxy measure of bioburden, it correlates closely with microbiological sampling in the literature [19]. Other proxy measures for bioburden such as the use of florescent dye were not included. Though the use of fluorescent techniques can show a measurable improvement in cleaning procedures, they do not necessarily demonstrated an impact on bioburden, depending on what is being used to remove the fluorescent dye. Therefore, studies that used improved cleaning practices or fluorescent marking as a proxy measure of bioburden were marked as “NA”.

All original studies were eligible if they were published before December 31, 2019, and if they measured the effect of an HEH intervention on HAI or patient colonization. Studies with an English abstract were eligible when published in English, French, German, or Spanish and only included if they were original research.

Studies were not eligible if they were conducted in vitro, did not include patient colonization or HAI as an outcome, were bundled with hand hygiene interventions, or were implemented during an outbreak. Outbreaks were excluded because outbreak management broadens the intervention, and it would not be possible to adjust for that effect. Complete structural rebuilds were excluded, because interventions such as renovating a building or replacing a plumbing system are not feasible HEH interventions in most contexts. There is also evidence that such interventions result in reduction of the studied pathogen for a limited time, after which the environment can become recolonized [20].

Interventions of interest were either mechanical, chemical, or they applied a human factors design. The standardized extraction forms included type of intervention, study title, authors, year of publication, study design, type of intervention(s), intervention(s), sample size or sample size proxy, control, microorganisms studied, outcome, whether the method is recommended for application by the authors, quality score and grade, reduction in bioburden, and comments.

Interventions were stratified into chemical, mechanical, human factors, and bundles of combining two or more of the aforementioned categories. Titles, abstracts and the full text of all potentially eligible studies were screened independently by at least two reviewers. Inclusions and exclusions were recorded following the PRISMA guidelines, and reasons for exclusion were detailed. Data were extracted by two authors. Any disagreement was resolved through discussion with a third author. Any missing data was requested from original study authors by email. Ethical approval was not required for this review.

As a wide variety of procedures and methodologies were identified, a descriptive analysis with a narrative synthesis was performed. Due to this heterogeneity, additional sub-group analyses by type of intervention, type of microorganism, and study quality were performed.

The study designs were divided into the following categories: randomized controlled trials (RCTs), quasi-experimental studies (prospective and retrospective), and before-and-after studies (prospective and retrospective). Sample sizes were categorized by ranges from less than 10 to more than 100′000 patients/patient-days/room cleanings. Presence of a study control was adjusted to include proxies for a control. The main confounding factors that were analyzed included hand hygiene compliance, antibiotic use, and the seasonality of certain HAI.

Available tools for analyzing study quality were assessed, and selected using the Strengthening the Reporting of Observational Studies in Epidemiology (STROBE) checklist for conducting observational studies which had been previously used for such a review [21, 22]. The STROBE checklist was, however, difficult to apply to some HEH interventions, in particular when a study had no control, its primary outcome was laboratory-based or based on bioburden measurements. We therefore also constructed a specifically-designed quality scoring system which included what the reviewers deemed the most important elements in the studies. Obviously, this scoring system is only meant to compare this specific list of studies and is not applicable in other contexts. After discussion in a working group, the following five elements were included in the quality assessment: study design, sample size, control, confounders, and issues with reporting. Among issues with reporting, conflict of interest (COI) was defined as minor if less than half of the authors disclosed a COI, such as having worked for industry as a consultant in the same field, and major if more than half of authors were funded by industry for the study.

Table 1 summarizes the quality scoring scale used in the review. Studies were graded from 0 to 20 points. “High quality” studies referred to studies that received an A or B grade according to the quality scale (Table 1). Some studies that ranked lower on the quality scale were well-performed, but simply not designed or powered to determine significant changes in patient colonization or HAI.

**Table 1:** Healthcare environmental hygiene intervention studies; quality scoring scale; systematic review

Scale	0	1	2	3	4
Study design	Before and after (retrospective, no control)	Before and after (prospective, no control)	Quasi experimental (retrospective, control)	Quasi experimental (prospective, control, not randomized)	Randomized controlled trial (prospective)
Sample size	Less than the above numbers/N/A	Over 10 patients/over 100 patient-days/over 100 room cleanings	Over 100 patients/over 1000 patient-days/over 1000 room cleanings	Over 1000 patients/over 10,000 patient-days/over 10,000 room cleanings	Over 10,000 patients/100,000 patient-days/100,000 room cleans
Control	No	N/A [1]	Proxy control/not well-executed	N/A	Yes
Adjusted for confounding factors	Not at all	N/A	Somewhat	N/A	Yes
Issues with reporting, including conflict of interest	Major COI^a^ and clear issues with data reporting	No/minor COI but clear issues with data reporting or major COI and minor issues with data reporting	No/minor COI but minor issues with data reporting or major COI and seemingly transparent data reporting	Minor COI and seemingly transparent data reporting	No COI and seemingly transparent data reporting

Studies were scored from a possible total of 20 points. Grade A was given for 16–20 points, B for 11–15 points, C for 6–10 points, and D for 0–5 points

*N/A* not available, *COI* conflict of interest

^a^Major COI referred to if over half of the study authors were funded by industry to conduct the study

## Findings

**Fig. 1:**
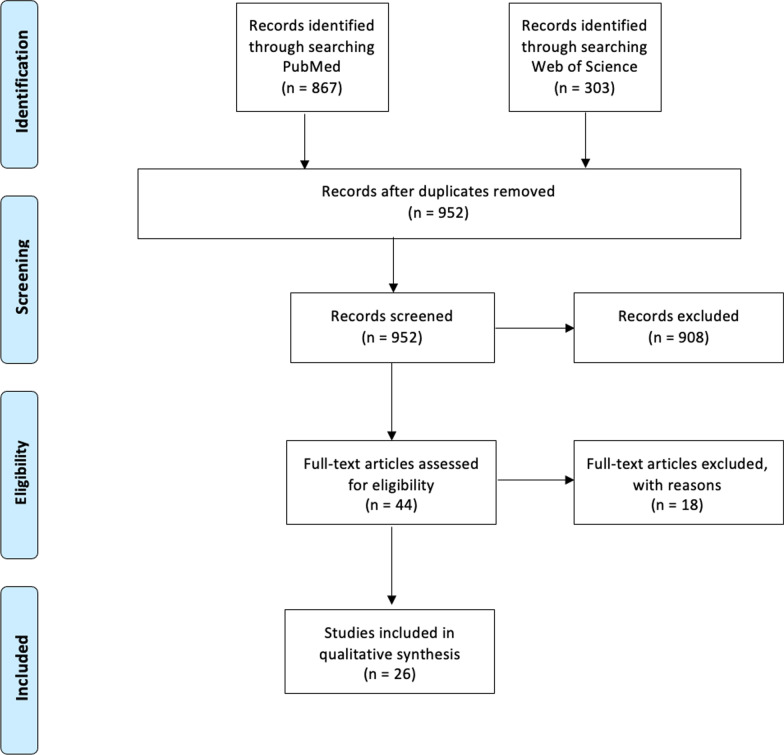
Effects of healthcare environmental hygiene interventions on healthcare-associated infections and patient colonization; Systematic review-PRISMA flow chart

Of the 952 retrieved and deduplicated studies, 44 were included for full-text review. A total of 26 studies were included in the final analysis (Fig. 1 and Table 2). Studies reported mechanical (n = 8) [23–30], chemical (n = 7) [31–37], human factors (n = 3) [38–40], and bundled interventions (n = 8) [41–48]. All of the studies that examined HAI only examined HAI in patients, not HCWs. Two studies were published before the year 1990 [25, 28], while the others (24/26) were published between 2013 and 2020. Of all of the 26 interventions, only five (19%) were not recommended for application by the study authors [23, 25, 30, 39, 42]. Among them, three were mechanical interventions [23, 25, 30], one was a human factors intervention [39], and one was a bundled intervention [42]. All of the chemical interventions were recommended for application by the study authors [31–37].

**Table 2:** Results of the environmental hygiene studies organized by type of intervention; systematic review; N = 26

Type of intervention	Study title	Year	Authors	Study design	Interventions	Sample size proxy	Sample size (patients)	Control	Microorganisms studied for colonization or HAI (same type)	Outcome: rate/reduction/cases	Method recom-mended*	Quality	Grade	Reduction in Bioburden	Comments
Mechanical	Protective isolation in a burns unit: the use of plastic isolators and air curtains [25]	1971	Lowbury et al	Prospective quasi experimental study	Isolators for burn patients (plastic, ventilated, air curtains both open and closed topped, with pre-filter and main filter)	NA	84	Open wards	*Coliform bacilli*, *P. aeruginosa*, *Proteus *sp., *S. aureus*	Lower incidence of infection with *P. aeruginosa* with intervention. *Proteus spp.* and miscellaneous *coliform bacilli* appeared on burns at least as often in isolators as in the open ward	No	12	B	Yes	Limited results for *P. aeruginosa*, other IPC^g^ measures are more important
Mechanical	Lack of nosocomial spread of Varicella in a pediatric hospital with negative pressure ventilated patient rooms [28]	1985	Anderson et al	Prospective before and after study	Negative pressure ventilation	NA	125	No	*H. zoster*, *V. zoster*	No cases of nosocomial spread in the new facility, with infected patients put in negative pressure rooms	Yes	6	C	NA	In a preceding study in an isolation facility without negative pressure ventilation, nosocomial infections occurred in 7 out of 41 susceptible patients who were on the same ward as two patients with chickenpox
Mechanical	Implementation and impact of ultraviolet environmental disinfection in an acute care setting [29]	2014	Haas et al	Retrospective before and after study	Pulsed Xenon UVC disinfection in the operating rooms (daily), dialysis unit (weekly), and terminal disinfection for all burn unit discharges	11,389 room cleans	NA	No	*C. difficile*, MDR Gram negative, MRSA, VRE^f^	Significant reduction in both incidence rates and HAI for VRE, MRSA, resistant gram-negative bacteria and *C. difficile*	Yes	9	C	NA	–
Mechanical	A Quasi-Experimental Study Analyzing the Effectiveness of Portable High-Efficiency Particulate Absorption Filters in Preventing Infections in Hematology Patients during Construction [26]	2016	Özen et al	Retrospective before and after study	HEPA^h^ filters	NA	413	No	Invasive fungal infections	Reduction of the HAI rates and reduction of invasive fungal infections in all of the patients following the installation of the HEPA filters. Intervention was significantly protective against IFI infection for specific groups of patients	Yes	10	C	NA	*Aspergillus* was mentioned in abstract but not specifically analyzed. But initial assessment was on the infection rates of both bacteria and fungi. Economic results should be taken cautiously because patients bills are unclear and significance of results depends on exchange rates
Mechanical	Impact of pulsed xenon ultraviolet light on hospital-acquired infection rates in a community hospital [27]	2016	Vianna et al	Prospective before and after study	Pulsed Xenon UVC terminal disinfection	> 4400 rooms	NA	No	*C. difficile*, MRSA, VRE	In non-ICU areas, significant reduction of *C. difficile*, no significant reduction of VRE, and significant increase of MRSA. In the ICU, reduction of all infections, but only a significant reduction for VRE	Yes, (though MRSA increased significantly)	5	D	NA	In non-ICU *only C. difficile* rooms received the intervention, which explains the results for the other pathogens
Mechanical	Pulsed-xenon ultraviolet light disinfection in a burn unit: Impact on environmental bioburden, multidrug-resistant organism acquisition and healthcare associated infections [30]	2017	Green et al	Prospective before and after study	Pulsed Xenon UVC^a^ terminal disinfection for *C. difficile* associated disease rooms, and some daily disinfection	653 occupied bed days	NA	No	*C. difficile*, Extended spectrum beta-lactamase Enterobacteriaceae, MDR^b^ *P.aeruginosa*, MRSA^c^, *S. maltophilia*	No statistically significant impact on HAI^d^ or MDR organisms acquisition. After intervention the ICU^e^ experienced along interval without HAI-*C. difficile* infection	No	8	C	Yes	Intervention period too short to really measure effect on colonization and HAI, study was not designed for this
Mechanical	Evaluation of an ultraviolet room disinfection protocol to decrease nursing home microbial burden, infection and hospitalization rates [24]	2017	Kovach et al	Prospective before and after study	Pulsed Xenon UVC terminal disinfection and shared living spaces disinfection	247	NA	No	N/A	Significant reductions in nursing home acquired relative to hospital-acquired infection rates for the total infections. Significant reduction of Hospitalizations for infection, with a notable reduction in hospitalization for pneumonia	Yes	6	C	Yes	-
Mechanical	Effectiveness of ultraviolet disinfection in reducing hospital-acquired *Clostridium difficile* and vancomycin-resistant Enterococcus on a bone marrow transplant unit [23]	2018	Brite et al	Prospective before and after study	Pulsed Xenon UVC disinfection and active surveillance	NA	579	No	*C. difficile*, VRE	No significant reduction in the incidence of VRE or *C. difficile* after the intervention	No	11	B	NA	-
Chemical	Impact of hydrogen peroxide vapor room decontamination on *Clostridium difficile* environmental contamination and transmission in a Healthcare setting [31]	2008	Boyce et al	Prospective before and after study	Gaseous hydrogen peroxide terminal disinfection and intensive disinfection in high incidence wards	NA	NA	No	*C. difficile*	Significant reduction of the nosocomial *C. difficile* incidence	Yes	8	C	Yes	Study was after an epidemic, once the strain had become endemic
Chemical	Implementation of hospital-wide enhanced terminal cleaning of targeted patient rooms and its impact on endemic *Clostridium difficile* infection rates [35]	2013	Manian et al	Retrospective before and after study	Gaseous hydrogen peroxide	196,313 patient-days	NA	No	*C. difficile*	Significant reduction of the nosocomial *C. difficile* associated disease rate between the preintervention period and intervention period	Yes	12	B	NA	-
Chemical	Copper surfaces reduce the rate of healthcare-acquired infections in the intensive care unit [37]	2013	Salgado et al	Randomized controlled trial	Copper alloy-coated objects	NA	431	Rooms without copper	MRSA, VRE	Significant lower rate of HAI and colonization in ICU rooms with intervention	Yes	10	C	Yes	Over half of intervention group not exposed to all copper surfaces, and over 13% of patients assigned to noncopper rooms were exposed to the intervention
Chemical	Use of a daily disinfectant cleaner instead of a daily cleaner reduced hospital-acquired infection rates [33]	2015	Alfa et al	Prospective quasi experimental study	Hydrogen peroxide disinfectant/detergent in disposable wipes	NA	NA	Similar hospital which only used detergent except for in *C. difficile* isolation rooms	*C. difficile*, MRSA, VRE	Significant reduction of all HAIs when cleaning compliance was high, and for VRE even when compliance was lower	Yes	13	B	NA	-
Chemical	Reduction in *Clostridium difficile* infection associated with the introduction of hydrogen peroxide vapour automated room disinfection [36]	2016	McCord et al	Retrospective before and after study	Gaseous hydrogen peroxide terminal disinfection	> 3000 patients room cleanings	NA	No	*C. difficile*	Significant reduction of the *C. difficile* infection rate	Yes	6	C	NA	Intervention is potentially cost saving
Chemical	Prospective cluster controlled crossover trial to compare the impact of an improved hydrogen peroxide disinfectant and a quaternary ammonium-based disinfectant on surface contamination and health care outcomes [32]	2017	Boyce et al	Randomized controlled trial	Daily cleaning with liquid hydrogen peroxide, feedback to staff	22,231 patient days	NA	Quaternary ammonium compounds (bleach for *C. difficile* rooms)	*C. difficile*, MRSA, VRE	No significant reduction of the composite colonization and infection outcome. (HAI and acquisition for VRE and MRSA, HAI for *C. difficile*)	Yes	17	A	Yes	Method recommended because surface contamination was also significantly lower
Chemical	Environmental disinfection with photocatalyst as an adjunctive measure to control transmission of methicillin-resistant Staphylococcus aureus: a prospective cohort study in a high-incidence setting [34]	2018	Kim et al	Before and after prospective	Photocatalyst antimicrobial coating (TiO2)	NA	621	No	*A. baumannii*, *C. difficile*, MRSA, VRE	Significant reduction in MRSA acquisition rate, and no significant reduction in the MRSA and *C. difficile* incidence rate. Significant reduction in incidence rate of hospital-acquired pneumonia. VRE and *A. baumannii* increased (not significantly)	Yes, for MRSA	11	B	Yes	-
Human factors	*Clostridium difficile* infection incidence: impact of audit and feedback programme to improve room cleaning [40]	2016	Smith et al	Retrospective before and after study	Online training, monitoring, weekly feedback	392,875 patient days	NA	No	*C. difficile*	Reduction of hospital-acquired *C. difficile* infection incidence following the intervention. After implementing the program, the rate of decline accelerated significantly	Yes	10	C	NA	Results may have been affected by confounding factors
Human factors	A Multicenter Randomized Trial to Determine the Effect of an Environmental Disinfection Intervention on the Incidence of Healthcare-Associated *Clostridium difficile* Infection [39]	2017	Ray et al	Randomized controlled trial	Training and monitoring of EVS personnel with feedback	1,683,928 patient days	NA	Disposable bleach wipes for daily and terminal disinfection, bleach, regular monitoring	*C. difficile*	No reduction in the incidence of healthcare-associated *C. difficile* infection during the intervention and postintervention periods	No	15	B	Yes	Environment was cleaner but no effect on *C. difficile* infection. No correlation between bioburden and HAI
Human factors	Environmental services impact on healthcare-associated *Clostridium difficile* reduction [38]	2019	Daniels et al	Retrospective quasi experimental design	Culture of safety with constructive feedback, education, auditing certifications, and accountability	52,290 patients days	NA	Hospitals where this system was already in use	*C. difficile*	Significant reduction in healthcare − associated *C. difficile* infections	Yes	15	B	NA	-
Bundle: chemical, human factors (minor)	Comparison of the effect of detergent versus hypochlorite cleaning on environmental contamination and incidence of *Clostridium difficile* infection [44]	2003	Wilcox et al	Prospective quasi experimental study	Hypochlorite with training	NA	NA	Detergent	*C. difficile*	Significant reduction in *C. difficile* infection associated with the use of hypochlorite in one of the study wards but not the other, where the *C. difficile* infection rate increased	Yes	11	B	Yes	-
Bundle: chemical, human factors	Controlling methicillin-resistant Staphylococcus aureus (MRSA) in a hospital and the role of hydrogen peroxide decontamination: an interrupted time series analysis [46]	2014	Mitchell et al	Retrospective before and after study	Gaseous hydrogen peroxide and liquid hydrogen peroxide disinfection; monitoring and feedback	3600 discharges, 32,600 swabs	NA	No	MRSA	Significant reduction of the incidence of MRSA colonization and infection after the introduction of the disinfectant	Yes	10	C	Yes	Study showed HEH can reduce infections, it does not prove superiority of hydrogen peroxide disinfectant, as it was compared to detergent
Bundle: chemical, human factors	A Successful Vancomycin-Resistant Enterococci Reduction Bundle at a Singapore Hospital [45]	2016	Fisher et al	Prospective before and after study	Training, gaseous hydrogen peroxide, workplace reminders (first part of study, before/during breakpoint), changed bleach cleaning solution, expanded surveillance, and automated alert system (later date, after reduction)	NA	270,000 (at least)	No	VRE	Significant reduction in the VRE rate	Yes	10	C	NA	Active surveillance, automated system and change in manual cleaning solution was only implemented well after the breakpoint in the reduction, so not causal for it.. Minimum sample size calculated form rate and total cases of VRE over 85 months is 270,000 patients)
Bundle: mechanical, chemical	Enhanced terminal room disinfection and acquisition and infection caused by multidrug-resistant organisms and *Clostridium difficile* (the Benefits of Enhanced Terminal Room Disinfection study): a cluster-randomised, multicentre, crossover study [48]	2017	Anderson et al	Randomized controlled trial	UVC terminal room disinfection ± Bleach	NA	21 395	Quaternary ammonium compounds(bleach for *C. difficile* rooms)	*C. difficile*, MDR *A. baumannii*, *S. aureus*, VRE	Significant reduction of composite risk of colonization for all organisms except *C. difficile*. For VRE, only bleach and bleach + UVC interventions caused significant reductions in HAI. No statistically significant decrease was seen when using UVC with bleach vs bleach alone (in *C. difficile* rooms)	Yes, when used with quaternary ammonium compounds (so recommended except for *C. difficile*)	19	A	Yes	Composite risk reduction is due to the major significant reduction for VRE
Bundle: chemical, mechanical, workflow	Control of endemic multidrug-resistant Gram-negative bacteria after removal of sinks and implementing a new water-safe policy in an intensive care unit [43]	2018	Shaw et al	Prospective before and after study	Deep cleaning and disinfection of drains and valves; antibacterial water filters in the taps; external cleaning with microfiber cloths and hypochlorite solution	35,909 patients-days	NA	No	*Klebsiella*, *Pseudomonas spp*.	Significant reduction of the incidence rates of MDR-Gram-negative bacteria after the intervention	Yes	10	C	NA	Different IPC interventions implemented during the study period (UVC, sink removal, antibiotic stewardship, environmental cleaning changes). No major changes in hand hygiene compliance
Bundle: human factors, mechanical, workflow	Reducing health care-associated infections by implementing separated environmental cleaning management measures by using disposable wipes of four colors [42]	2018	Wong et al	Prospective before and after study	Training, education and awareness regarding cleaning and 4 color coded reusable wipes	NA	635	Reusable wipes soaked with hypochlorite solution, visual inspection	*C. difficile*, MRSA, VRE	No reduction in HAI density after intervention, but it was during the follow-up period	No	7	C	Yes	Calling the wipes "disposable" is misleading, wipes were disposed after a number of uses depending on the color/environment
Bundle: chemical (minor), human factors, mechanical (minor)	An environmental cleaning bundle and health-care-associated infections in hospitals (REACH): a multicentre, randomised trial [47]	2019	Mitchell et al	Randomized controlled trial	Training, auditing, feedback, implementation of enhanced cleaning practices, and the incorporation of disposable wipes	3,534,439 patient bed-days	NA	Periods where hospitals were not implementing the bundle	*C. difficile*, *S. aureus*, VRE	Significant reduction of VRE infections. No significant changes in the incidence of *S. aureus* bacteremia and of *C. difficile* infections	Yes, for VRE	19	A	NA	Not all hospitals used the wipes, and not all disinfected appropriately for *C. difficile*, which explains the results
Bundle: human factors, workflow	Implementation of human factors engineering approach to improve environmental cleaning and disinfection in a medical center [41]	2020	Hung et al	Prospective before and after study	Education, feedback, redesigned workflow of terminal cleaning and disinfection, a regular method of bleach dilution, and a checklist-form reminder)	NA	NA	No	Carbapenem-resistant *A. baumannii* complex, MRSA, VRE	Significant reduction in total MDRO colonization, but no reduction in HAI	Yes	5	D	Yes	Very few results on HAI, results are technically correlation. No information on specific pathogens for HAI, no adjustment for confounding factors. Authors recommend measures although HAI rates did not improve

*Recommended by the study authors, ^a^*UVC* ultraviolet-C light, ^b^*MDR* multidrug resistant, ^c^*MRSA* multidrug-resistant *S. aures;* ^d^*HAI* Healthcare-associated infections; ^e^*ICU* Intensive Care Unit; ^f^*VRE* vancomycin-resistant enterococci, ^g^*IPC* infection prevention and control, ^h^*HEPA* high efficiency particulate air (filter)

Five studies were RCTs [32, 37, 39, 47, 48]. The remaining studies had prospective quasi-experimental designs (n = 3) [25, 33, 44], retrospective quasi-experimental design (n = 1) [38], prospective before-and-after designs (n = 11) [23, 24, 27, 28, 30, 31, 34, 41–43, 45], and retrospective before-and-after designs (n = 6) [26, 29, 35, 36, 40, 46]. In total, only 31% (8/26) studies had a true control [25, 32, 37, 39, 42, 44, 47, 48].

Over half (15/26, 58%) of the studies demonstrated a significant decrease in patient colonization or HAI following the chosen intervention for all microorganisms tested [24, 26, 29, 31, 33, 35–38, 40, 41, 43–46]. In one study, the reduction was not significant for all patient groups [26]. If additional interventions that demonstrated a reduction in all microorganisms tested were included, whether significant or not, this increased to 69% [23, 28, 32]. If the additional interventions that demonstrated a reduction in at least one of the microorganisms tested (significant or not) were included, this increased to 88% [25, 27, 34, 47, 48].

### Analysis by type of intervention (Table 2)

Of the eight studies that implemented mechanical interventions [23–30], 63% (5/8) reported statistically significant reductions in HAI or colonization for at least one tested microorganism [24–27, 29]. When all mechanical interventions showing any reduction in at least one of the microorganisms tested were included, including those not statistically significant, this increased to 88% (7/8) [23, 48]. Two of the three studies that implemented human factors interventions [38–40], showed a statistically significant reduction in HAI or colonization for all microorganisms tested [38, 40]. The remaining study demonstrated no reduction [39]. Of the seven studies that implemented chemical interventions [31–37], 6 (86%) demonstrated statistically significant reductions for at least one of the microorganisms tested [31, 33–37]. If all the interventions that demonstrated a reduction (not significant) in all microorganisms tested were considered, this increased to 100%. Eight studies implemented bundled interventions, and 88% (7/8) demonstrated statistically significant reductions in HAI or colonization for at least one of the microorganisms tested [41, 43–48], although the study by Anderson et al. [48] only demonstrated significant reduction in one of the two test wards. The remaining study demonstrated no reduction [42].

Sub-group analyses were conducted for the most frequently implemented interventions (Table 3): ultraviolet-C light (UVC), hydrogen peroxide (both liquid and gaseous), and human factors. UVC interventions were implemented in six studies [23, 24, 27, 29, 30, 48]. Of these, one study was bundled [48]. The interventions were recommended for application by the authors in four (67%) of the studies [24, 27, 29, 48]. Reductions in colonization/HAI were significant in those same four studies, though not for all microorganisms tested [27, 48].

**Table 3:** Healthcare environmental hygiene interventions according to the individual type of intervention; systematic review

Interventions	Number	Type
UVC^a^ [23, 24, 27, 29, 30, 48]	6	Mechanical
Training, monitoring, feedback [38–40]	3	Human factors
Gaseous hydrogen peroxide [31, 35, 36]	3	Chemical
Liquid hydrogen peroxide [32, 33]	2	Chemical
Negative pressure ventilation system [28]	1	Mechanical
Isolators and air curtains [25]	1	Mechanical
HEPA^a^ filters [26]	1	Mechanical
TiO_2_ antimicrobial surface coating [34]	1	Chemical
Copper antimicrobial surface coating [37]	1	Chemical
Training and education and color-coded wipes [42]	1	Bundle: human factors and mechanical
Training and education, monitoring and feedback and workflow changes [41]	1	Bundle: human factors and workflow
External cleaning with microfiber and hypochlorite, water filters, and deep cleaning [43]	1	Bundle: chemical and mechanical and workflow
Hypochlorite with training [44]	1	Bundle: chemical and human factors (minor)
Gaseous hydrogen peroxide, change in bleach cleaning solution, training and education, monitoring and feedback, increased surveillance, and workplace reminders [45]	1	Bundle: chemical and human factors
Gaseous hydrogen peroxide, liquid hydrogen peroxide, monitoring and feedback [46]	1	Bundle: chemical and human factors
Training and education, monitoring and feedback, enhanced cleaning practices, disposable wipes [47]	1	Bundle: human factors, chemical (minor), mechanical (minor)

^a^*UVC* ultraviolet-C light, *HEPA* high efficiency particulate air, *TiO*_2_ titanium dioxide

Five studies assessed the implementation of gaseous hydrogen peroxide [31, 35, 36, 45, 46]; two were bundled interventions [45, 46]. The interventions were recommended for application by authors in all studies, and all reductions were statistically significant. Three studies assessed liquid hydrogen peroxide [32, 33, 46]. The interventions were recommended in all studies, and the reductions in colonization/HAI were statistically significant in two studies [33, 46].

Human factors studies encompassed all interventions that included training and education, monitoring and feedback, and promotion of institutional safety climate. Nine studies assessed the implementation of human factors [38–42, 44–47]; six were bundled interventions [41, 42, 44–47]. The interventions were recommended by the authors in 78% (7/9) of the studies [38, 40, 41, 44–47], though one only recommended it for VRE [47]. Reductions in colonization/HAI were significant in those same studies.

One study performed a cost analysis. The installation of high efficiency particulate air (HEPA) filters was found to decrease the cost per patient; it is to note that these findings were significant in both $ and €, but did not reach the threshold for significance in Turkish Lira [26]. Another article suggested that gaseous hydrogen peroxide decontamination was cost-effective for *C. difficile*, based on the estimated minimum cost of nosocomial *C. difficile* infection per year [36].

### Analysis by microorganism (Table 2)

Half of the studies (13/26) observed the impact of an intervention on methicillin-resistant *Staphylococcus aureus* (MRSA) and/or *S. aureus* [25, 27, 29, 30, 32–34, 37, 41, 42, 46–48]. Of these, 62% (8/13) were recommended for application by the study authors [29, 32–34, 37, 41, 46, 48]. One study that recommended the intervention compared a disinfectant to a detergent [46], and one which did not recommend the intervention was not powered to demonstrate a reduction in HAI [30]. 46% of the interventions (6/13) demonstrated a significant decrease in HAI/colonization [29, 33, 34, 37, 41, 46]. In one study that did not, the rate of MRSA infection increased significantly, which is unsurprising, as the intervention was only implemented in *C. difficile* rooms in the arm of the study with the increase [27].

Sixty-five percent of studies (17/26) observed the impact of an intervention on *C. difficile* [23, 27, 29–36, 38–40, 42, 44, 47, 48]. Among these, 59% of the interventions (10/17) were recommended for application by the study authors [27, 29, 31–33, 35, 36, 38, 40, 44]. Of the seven studies that were not recommended, one was not powered to be able to show a reduction in HAI and not all hospitals disinfected appropriately for *C. difficile* in another [30, 47]. Fifty-three percent of the interventions (9/17) demonstrated a significant decrease in HAI/colonization [27, 29, 31, 33, 35, 36, 38, 40, 44].

Forty-six percent of studies (12/26) observed the impact of a HEH intervention on VRE [23, 27, 29, 32–34, 37, 41, 42, 45, 47, 48]. Of these, 75% (9/12) recommended the intervention [27, 29, 32, 33, 37, 41, 45, 47, 48]. 58% of studies (7/12) demonstrated a significant decrease in HAI/colonization [29, 33, 37, 41, 45, 47, 48]. One study demonstrated that the intervention reduced the rate of colonization but not of HAI [41]. One study demonstrated that VRE colonization was reduced even when compliance to the intervention was lower than necessary for significantly reducing other pathogens [33].

Seven studies assessed the effect of interventions on Gram negative bacteria [25, 29, 30, 34, 41, 43, 48]. Three studies observed the impact of an intervention on *A. baumannii* (including carbapenem-resistant and multidrug-resistant strains) [34, 41, 48], and three on Pseudomonas (two on *P. aeruginosa* and one on *Pseudonomas* spp.) [25, 30, 43]. *Klebsiella*, extended spectrum beta-lactamase *Enterobacteriaceae*, *S. maltophilia*, *Proteus* sp. and coliform bacilli were each analyzed by only one study [25, 30, 43]. Fifty-seven percent of interventions (4/7) were recommended for application by the authors, each of which demonstrated a significant decrease in HAI/colonization [29, 41, 43, 48]. One older study [28] evaluated the role of negative air pressure rooms to prevent *Varicella zoster* and *Herpes zoster* infection. Although statistical significance was not calculated, there were no new cases after the intervention and the method was recommended by the authors [28]. Another study demonstrated the effect of air control to prevent invasive fungal infections during construction and showed an effect among oncology-haematology patients [26].

### Analysis by quality (Table 4)

**Table 4:** Quality scoring of included studies; systematic review; N = 26

Study title	Study design	Sample size	Control	Adjusted for confounding factors	Conflict of interest and reporting	Final grade
Prospective cluster controlled crossover trial to compare the impact of an improved hydrogen peroxide disinfectant and a quaternary ammonium-based disinfectant on surface contamination and health care outcomes [32]	4	2	4	4	3	A
Enhanced terminal room disinfection and acquisition and infection caused by multidrug-resistant organisms and *Clostridium difficile* (the Benefits of Enhanced Terminal Room Disinfection study): a cluster-randomised, multicentre, crossover study [48]	4	4	4	4	3	A
An environmental cleaning bundle and health-care-associated infections in hospitals (REACH): a multicentre, randomised trial [47]	4	4	4	4	3	A
Effectiveness of ultraviolet disinfection in reducing hospital-acquired *Clostridium difficile* and vancomycin-resistant Enterococcus on a bone marrow transplant unit [23]	1	2	0	4	4	B
Environmental disinfection with photocatalyst as an adjunctive measure to control transmission of methicillin-resistant Staphylococcus aureus: a prospective cohort study in a high-incidence setting [34]	1	2	0	4	4	B
Comparison of the effect of detergent versus hypochlorite cleaning on environmental contamination and incidence of *Clostridium difficile* infection [44]	3	0	4	2	2^a^	B
Protective isolation in a burns unit: the use of plastic isolators and air curtains [25]	3	1	4	2	2^a^	B
Implementation of hospital-wide enhanced terminal cleaning of targeted patient rooms and its impact on endemic *Clostridium difficile* infection rates [35]	0	4	0	4	4	B
Use of a daily disinfectant cleaner instead of a daily cleaner reduced hospital-acquired infection rates [33]	3	0	2	4	4	B
Environmental services impact on healthcare-associated *Clostridium difficile* reduction [38]	2	3	2	4	4	B
A Multicenter Randomized Trial to Determine the Effect of an Environmental Disinfection Intervention on the Incidence of Healthcare-Associated *Clostridium difficile* Infection [39]	4	4	4	0	3	B
Lack of nosocomial spread of Varicella in a pediatric hospital with negative pressure ventilated patient rooms [28]	1	1	2	0	2^b^	C
Evaluation of an ultraviolet room disinfection protocol to decrease nursing home microbial burden, infection and hospitalization rates [24]	1	2	0	0	3	C
Reduction in *Clostridium difficile* infection associated with the introduction of hydrogen peroxide vapour automated room disinfection [36]	1	2	0	0	3	C
Reducing health care-associated infections by implementing separated environmental cleaning management measures by using disposable wipes of four colors [42]	1	2	0	0	4	C
Impact of hydrogen peroxide vapor room decontamination on *Clostridium difficile* environmental contamination and transmission in a healthcare setting [31]	1	0	0	4	3	C
Pulsed-xenon ultraviolet light disinfection in a burn unit: Impact on environmental bioburden, multidrug-resistant organism acquisition and healthcare associated infections [30]	1	1	0	2	4	C
Implementation and impact of ultraviolet environmental disinfection in an acute care setting [29]	0	3	0	2	4	C
A Successful Vancomycin-Resistant Enterococci Reduction Bundle at a Singapore Hospital [45]	1	4	0	2	3	C
Controlling methicillin-resistant Staphylococcus aureus (MRSA) in a hospital and the role of hydrogen peroxide decontamination: an interrupted time series analysis [46]	0	2	0	4	4	C
A Quasi-Experimental Study Analyzing the Effectiveness of Portable High-Efficiency Particulate Absorption Filters in Preventing Infections in Hematology Patients during Construction [26]	0	2	0	4	4	C
Copper surfaces reduce the rate of healthcare-acquired infections in the intensive care unit [37]	4	2	2	2	0	C
Control of endemic multidrug-resistant Gram-negative bacteria after removal of sinks and implementing a new water-safe policy in an intensive care unit [43]	1	3	0	2	4	C
*Clostridium difficile* infection incidence: impact of audit and feedback programme to improve room cleaning [40]	0	4	0	2	4	C
Implementation of human factors engineering approach to improve environmental cleaning and disinfection in a medical center [41]	1	0	0	0	4	D
Impact of pulsed xenon ultraviolet light on hospital-acquired infection rates in a community hospital [27]	1	2	0	0	2	D

^a^Information on COI not complete, with appropriate complementary information, this could be a 4

^b^Information on COI not complete, with appropriate complementary information, this could be a 4

The quality scoring system (Table 1) considered study design, sample size, whether there was a control, how the study adjusted for confounding factors, and issues in reporting. Table 4 shows the detailed quality scoring system results for the 26 studies. Forty-two percent of the studies (11/26) were considered to be of high-quality (grade A or B, Table 4). All studies that were of quality “A” and 1 study of quality “B” were RCTs [32, 39, 47, 48]. 27% of high-quality study interventions (3/11) were not recommended for application by the authors [23, 25, 39]. The interventions in 64% (7/11) of these studies significantly reduced colonization/HAI [33–35, 38, 44, 47, 48]. In 43% (3/7) of these studies, the reduction was only significant for specific bacteria [34, 44, 47]. Fifty-eight percent of the studies (15/26) were of lower quality (grade of C or D, Table 4). Eighty-six percent of these (13/15) significantly reduced colonization/HAI [24, 26–29, 31, 36, 37, 40, 41, 43, 45, 46]. In one of these studies, the reduction was only significant for specific bacteria [27].

A further analysis was conducted which included only the higher quality studies that used a true control, and the most commonly studied microorganisms (*S. aureus*, *C. difficile*, and VRE), in order to assess whether there was a significant reduction per pairing of each microorganism and intervention (Table 5). This resulted in 15 of pairings from five studies [32, 39, 44, 47, 48]. The distribution included five interventions for each *S. aureus*, *C. difficile*, and VRE. Eighty-seven percent of the pairings (13/15) demonstrated a reduction in colonization or HAI [32, 44, 47, 48], but only 27% of them (4/15) demonstrated a significant reduction in patient colonization or HAI [44, 47, 48]. Studies were too heterogenous to perform any kind of metanalysis, and in those high quality studies, no two interventions on the same microorganism were comparable. Future studies in the field should aim to calculate sample sizes and be adequately powered to be able to demonstrate such reductions.

**Table 5:** Effects of healthcare environmental hygiene interventions on healthcare-associated infections and patient colonization

Author	Micro-organism	Intervention	Total reduction	Significant reduction	Effect of the HEH intervention
Wilcox et al. [44]	*C. difficile*	Hypochlorite	Yes	Yes	Rate of colonization: NA Rate of HAI for both wards combined: 12.4–10 Unit of measure: 100 admissions RR: NA CI: NA *P* value: < 0.05
Anderson et al. [48]	*C. difficile*	UV	Yes	No	Rate of colonization and rate of HAI (combined): 31.6–30.4 Unit of measure: 10,000 exposure days RR: 1.0 CI: 95%CI 0.57–1.75 *P* value: 0.997
Boyce et al. [32]	*C. difficile*	Liquid hydrogen peroxide	Yes	No	Rate of colonization and rate of HAI (combined): 1.0–0.56 Unit of measure: number of cases per 1000 patient days RR: NA CI: NA *P* value: NA Composite outcome (colonization + HAI rate of all microbes): 10.3–8.0 incidence rate ratio 0.77; *P* = 0.068; 95%CI 0.579–1.029
Ray et al. [39]	*C. difficile*	Training, monitoring and feedback	No	No	No data available for the intervention period. rate of colonization: NA rate of HAI for preintervention period only (intervention vs. control hospitals): 5.6–5.8 Unit of measure: 10,000 patient days RR: NA CI: NA *P* value: 0.8
Mitchell et al. [47]	*C. difficile*	Bundle	No	No	Rate of colonization: NA Rate of HAI: 2.34–2.52 Unit of measure: 10,000 occupied bed-days RR: 1.07 CI: 95%CI 0·88–1.30 *P* value: 0.4655
Anderson et al. [48]	*S. aureus*	UV	Yes	No	Rate of colonization and rate of HAI (combined): 50.3–36.5 Unit of measure: 10,000 exposure days RR: 0.78 CI: 95%CI 0.58–1.05 *P* value: 0.104
Anderson et al. [48]	*S. aureus*	Bleach	Yes	No	Rate of colonization and rate of HAI (combined): 50.3–48.2 Unit of measure: 10,000 exposure days RR: 1.00 CI: 95%CI 0.82–1.21 *P* value: 0.967
Anderson et al. [48]	*S. aureus*	Bundle: UV + bleach	Yes	No	Rate of colonization and rate of HAI (combined): 50.3–46.9 Unit of measure: 10,000 exposure days RR: 0.97 CI: 95%CI 0.78–1.22 *P* value: 0.819
Boyce et al. [32]	*S. aureus* (MRSA)	Liquid hydrogen peroxide	Yes	No	Rate of colonization and rate of HAI (combined): 2.79–1.96 Unit of measure: number of cases per 1,000 patient days RR: NA CI: NA *P* value: NA Composite outcome (colonization + HAI rate of all microbes): 10.3–8.0 incidence rate ratio 0.77; *P* = 0.068; 95%CI 0.579–1.029
Mitchell et al. [47]	*S. aureus*	Bundle	Yes	No	Rate of colonization: NA rate of HAI: 0.97–0.80 Unit of measure: 10,000 occupied bed-days RR: 0.82 CI: 95%CI 0.60–1.12 *P* value:0.2180
Anderson et al. [48]	VRE	UV	Yes	No	Rate of colonization and rate of HAI (combined): 63.4–29.4 Unit of measure: 10,000 exposure days RR: 0.41 CI: 95%CI 015–1.13 *P* value: 0.084
Anderson et al. [48]	VRE	Bleach	Yes	Yes	Rate of colonization and rate of HAI (combined): 63.4–31.9 Unit of measure: 10,000 exposure days RR: 0.43 CI: 95%CI 0.19–1.00 *P* value: 0.049
Anderson et al. [48]	VRE	Bundle: UV + bleach	Yes	Yes	Rate of colonization and rate of HAI (combined): 63.4–39.0 Unit of measure: 10,000 exposure days RR: 0.36 CI: 95%CI 0.18–0.70 *P* value: 0.003
Boyce et al. [32]	VRE	Liquid hydrogen peroxide	Yes	No	Rate of colonization and rate of HAI (combined): 6.6–5.49 Unit of measure: number of cases per 1,000 patient days RR: NA CI: NA P value: NA Composite outcome (colonization + HAI rate of all microbes): 10.3–8.0 incidence rate ratio 0.77; *P* = 0.068; 95%CI 0.579–1.029
Mitchell et al. [47]	VRE	Bundle	Yes	Yes	Rate of colonization: NA rate of HAI: 0.35–0.22 Unit of measure: 10,000 occupied bed-days RR: 0.63 CI: 95%CI 0.41–0.97 *P* value: 0.0340

Studies were selected if they had a quality rating of “A” or “B” (Table 4), used a control and if they studied the three most commonly-examined microorganisms

Significance of individual experiments on commonly studied microorganisms per method of intervention; systematic review

### Bioburden (Table 6)

**Table 6:** Relation between the reduction in environmental bioburden and patient colonization or healthcare- associated infection following an environmental hygiene intervention; systematic review

Authors	Interventions	Bioburden measurement: ATP/culture	Microorganisms with significant reduction for colonization	Microorganisms with significant reduction for HAI	Total microorganisms evaluated for colonization or HAI
Lowbury et al. [25]	Isolators for burn patients	Settle plates of *S. aureus*	NA	NA	*Coliform bacilli*, *P. aeruginosa*, *Proteus *sp., *S. aureus*
Wilcox et al. [44]	Hypochlorite, training	Culture of *C. difficile*	NA	*C. difficile*	*C. difficile*
Boyce et al. [31]	Gaseous hydrogen peroxide (HPV)	Culture of *C. difficile*	No	*C. difficile*	*C. difficile*
Salgado et al. [37]	Copper alloy-coating	Culture of MRSA, VRE, *A. baumanni, P. aeruginosa*, *E. coli*	Composite (MRSA, VRE)	Composite (MRSA, VRE)	MRSA, VRE
Mitchell et al. [46]	Gaseous HP (HPV) and liquid HP; monitoring, feedback	Culture of MRSA	MRSA	MRSA	MRSA
Anderson et al. [48]	UV-C terminal room disinfection ± Bleach	Culture of MRSA, VRE, *C. difficile*, MDR *A. baumannii*	VRE and composite (MDR *A. baumannii*, *S. aureus*, VRE)	VRE for bleach and bleach + UV arms	*C. difficile*, MDR *A. baumannii*, *S. aureus*, VRE
Boyce et al. [32]	Liquid HP, feedback	Culture of MRSA, VRE, *C. difficile*	No	No	*C. difficile*, MRSA, VRE
Green et al. [30]	Pulsed Xenon UV	Culture of (Bacillus spp., coagulase negative staphylococci, Micrococcus spp., Corynebacterium aurimucosum, Dietzia cinnamea, Moraxella osloensis, Sphingomonas paucimobilis, mold, other presumed environmental isolates (listed as large Gram-positive cocci, Gram-positive rods, or unknown/not described); gram negative rod, MDRO, *C. difficile)*	No	No	*C. difficile*, ESBL *Enterobacteriacae*, MDR P.aeruginosa, MRSA, *S. maltophilia*
Kovach et al. [24]	Pulsed Xenon UV	ATP; culture of gram-positive cocci or rod, gram-positive bacilli	No	NA	NA
Ray et al. [39]	Training, monitoring, feedback	ATP; culture of *C. difficile*	No	No	*C. difficile*
Kim et al. [34]	Photocatalyst antimicrobial coating (TiO2)	Culture of Staphylococcus spp., Bacillus spp.	MRSA	No	*A. baumannii*, *C. difficile*, MRSA, VRE
Wong et al. [42]	Training, education, color-coded wipes	ATP	NA	No	*C. difficile*, MRSA, VRE
Hung et al. [41]	Education, feedback, redesigned workflow	ATP; aerobic colony counts (ACC) of unknown micro-organisms	Composite (CRABC, MRSA, VRE)	No	CRABC, MRSA, VRE

*ATP* adenosine triphosphate, *CRBAC* Carbapenem-resistant *Acinetobacter baumannii* complex, *MRSA* multidrug-resistant *S. aureus*, *VRE* vancomycin-resistant enterococci, *N/A* not available

Fifty percent (13/26) of studies observed the impact of HEH interventions on environmental bioburden [24, 25, 30–32, 34, 37, 39, 41, 42, 44, 46, 48]. 100% of them demonstrated that the interventions decreased environmental bioburden. Over half (7/13) of the studies demonstrated bioburden reductions paralleled directly with a significant reduction in colonization/HAI for at least one of the microorganisms of interest [31, 34, 37, 41, 44, 46, 48].

## Interpretation

This systematic review demonstrated that interventions in environmental hygiene were often associated with a reduction in HAI in a seemingly causal way. Over half of studies demonstrated a significant decrease in colonization or HAI for all of the microorganisms tested. These results are indicative of the importance of environmental hygiene in patient safety.

There were major issues with both the heterogeneity of the interventions and the settings, as well with the quality in a number of the studies, hence the sub analyses. There are relatively few high quality studies in HEH compared to other fields, and even the use of RCTs in the field is exceedingly rare [11]. One high-quality study [49] in particular would have been useful for the review, but was excluded due to a hand hygiene intervention. Often, the primary study outcome evaluated environmental bioburden. Though HAI or patient colonization was a secondary outcome obtained from hospital data, these studies were not necessarily designed and powered to analyze this outcome. The measurable impact of HEH is likely to be more apparent if future studies are sufficiently powered.

Most of the studies that did not show a statistically significant reduction in HAI or patient colonization nonetheless recommended their interventions for application because they did greatly reduce environmental bioburden [28, 32, 38]. Though eight studies had controls [25, 32, 37, 39, 42, 44, 47, 48], many had before-and-after study designs [23, 24, 26–31, 34–36, 40, 41, 43, 45, 46], and thus did not implement appropriate controls. Two used similar institutions as “proxy” controls [33, 38]. Often, studies used the baseline rate of colonization or HAI before the intervention was implemented, and attempted to account for some confounding factors such as hand hygiene, antimicrobial use, and seasonality of the diseases of interest. In retrospect, it may have been more useful to only analyze more recent studies, because the two that were published before 2000 [25, 28] (in 1971 and 1985, respectively) were exploring different research questions and microorganisms.

The success of the interventions also depended on which microorganisms were studied, and how successfully or not specific pathogens spread through the healthcare environment. For example, VRE, known to spread through the environment, was sometimes more successfully reduced than pathogens known to frequently spread through hands from patient to patient. One study [26] testing air filters gave further support to the fact that not all microorganisms are able to be transmitted by air, unlike what some manufacturers claim.

Considering the subset analysis targeted on specific pathogens, it is important to note that not all studies were designed to demonstrate the efficacy of a particular intervention on colonization/HAI, as this was not always the primary outcome. Some interventions were recommended by the authors for application because they demonstrated a significant reduction in some pathogens but not in others. Though these outcomes were often coupled with a significant decrease in environmental bioburden, some studies were not sufficiently powered to demonstrate that the reduction was statistically significant.

Overall, the selected studies were very heterogenous; both in terms of the types of interventions and their quality. The review attempts to address some of these limitations by performing subset analyses. However, the results reflect the reality of this field; there is a significant amount of work left to be done. Though COVID-19 has generated an increased global interest in HEH, the bulk of newer studies were performed during a pandemic, and were not included in this review, as interventions conducted during outbreak situations were excluded.

## Conclusion

Although more high quality studies are needed, this review demonstrates a strong relation between interventions to improve HEH and a reduction in both environmental bioburden and in patient colonization or HAI. Optimal HEH practices are an integral part of patient safety and a key component to improving infection prevention and control. Healthcare institutions may be able to lower their HAI rates by improving HEH practices. The domain of HEH deserves further and better-designed field research.

## Supplementary Information

**Additional file 1:** Full search strategy for the systematic review on the impact of environmental hygiene interventions on healthcare-associated infections and patient colonization.

## Data Availability

The datasets generated and/or analysed during the current study are available in PROSPERO repository, https://www.crd.york.ac.uk/PROSPEROFILES/204909_STRATEGY_20200908.pdf. All other data are all data generated or analysed during this study are included in this published article and its Additional file 1.

## Abbreviations

ATP: Adenosine triphosphate
COI: Conflict of interest
HAI: Healthcare-associated infections
HCWs: Healthcare workers
HEH: Healthcare environmental hygiene
HEPA: High efficiency particulate air (filter)
ICU: Intensive care unit
IPC: Infection prevention and control
MDR: Multidrug resistant
MRSA: Multidrug-resistant *S. aureus*
PRISMA: Preferred Reporting Items for Systematic Reviews and Meta-Analysis
QAC: Quaternary ammonium compound
RCT: Randomized controlled trial
UVC: Ultraviolet-C light
VRE: Vancomycin-resistant enterococci
WHO: World Health Organization
