# Supplementary material for: Impact of environmental hygiene interventions on healthcare-associated infections and patient colonization: a systematic review
Source: Antimicrob Resist Infect Control. 2022 Feb 19;11:38. doi: 10.1186/s13756-022-01075-1 (PMC8857881; doi:10.1186/s13756-022-01075-1)
Supplement: Supplementary file 1 — Additional file 1: Full search strategy for the systematic review on the impact of environmental hygiene interventions on healthcare-associated infections and patient colonization. [file 13756_2022_1075_MOESM1_ESM.pdf]

("Cross Infection"[MeSH] OR ("Catheter-Related Infections"[Mesh] OR ("Pneumonia, Ventilator-Associated"[Mesh] OR (VAP OR (Ventilator Associated Event* OR ("Surgical Wound Infection"[Mesh] OR ("Hospital acquired infection*" OR (“Hospital infection*” OR (VRE OR ("Healthcare associated infection*" OR ("Nosocomial infection*" OR (“Catheter related infection*” OR ("Catheter associated infection*" OR ("Central line associated bloodstream infection*" OR (CLABSI OR (CRBSI OR (“Catheter related bloodstream infection*” OR (CAUTI OR ("Catheter associated bloodstream infection*" OR ("Catheter associated urinary tract infection*" OR ("Surgical site infection*" OR ("Post-operative infection*" OR (CPE OR (CRE OR ("Carbapenem-Resistant Enterobacteriaceae"[Mesh] OR (carbapenemase OR ("Methicillin-Resistant Staphylococcus aureus"[Mesh] OR (MRSA OR ("Post-surgical infection*" OR ("clostridium difficile"[MeSH] OR (Legionellaceae[MeSH] OR (Pseudomonas[MeSH] OR (“Staphylococcus epidermidis”[MeSH] OR (“Staphylococcus aureus”[MeSH] OR (Enterobacteriaceae[MeSH] OR (ESBL OR (“Extended Spectrum Beta Lactamase” OR (“Acinetobacter baumannii”[MeSH] OR (“Acinetobacter Infections“[MeSH] OR (Legionellosis[MeSH] OR (“Gram-Negative Bacterial Infections”[MeSH] OR (“Gram-Positive Bacterial Infections”[MeSH] OR (“Staphylococcal Infections”[MeSH] OR (“Enterococcus faecium”[MeSH] OR (“Vancomycin-Resistant Enterococci”[MeSH] OR (“Enterococcus faecalis”[MeSH] OR (“Pseudomonas Infections”[MeSH] OR (“Influenza, Human”[MeSH] OR (“Hepatitis A”[MeSH] OR (“Hepatitis A virus”[MeSH] OR (“Hepatitis B”[MeSH] OR (“Hepatitis A Virus, Human”[MeSH] OR (HIV[MeSH] OR (Norovirus[MeSH] OR (“Stenotrophomonas maltophilia”[MeSH] OR (Tuberculosis[MeSH] OR (Candida[MeSH] OR (“Serratia marcescens”[MeSH] OR (“Serratia Infections”[MeSH] OR (“Disease transmission, infectious”[MeSH] OR (colonis* OR (coloniz* OR (HAI))) AND (Intervention* OR (“waste management” OR (“ward cleaning” OR (bundle OR (“Cleaning bundle*” OR (“Hygiene bundle*” OR (Training* OR (Hygiene OR (“Water treatment” OR (“Water filt*” OR (“Sanitation” OR (“Air sanitation” OR (“Air filt*“ OR (Disinfection OR (Disinfectant OR (“Air purification“ OR (“Air treatment“ OR (“Waste management” OR (“Waste treatment” OR (IPC OR (“infection prevention” OR (Sterilization OR (“Instrument reprocessing”))))))))))))))))))))))) AND ("Health Facility Environment"[MeSH] OR (“high touch surfaces” OR (“multi touch surfaces” OR (“environmental hygiene” OR (“Hospital environment” OR (“healthcare environment”)))))) AND (patient OR (patients OR (“healthcare worker*” OR (HCW))))
